# Comparative Insights into Enzymatic and High-Temperature Steam Treatment of *Glycyrrhiza uralensis* Fisch. Stems: Structural Remodeling, Flavonoid Transformation and Antioxidant Enhancement

**DOI:** 10.3390/foods15183315

**Published:** 2026-09-19

**Authors:** Man Zhou, Qier Mu, Na Liu, Yuan Sun, Xiaoping An, Jingwei Qi

**Affiliations:** 1 College of Animal Science, Inner Mongolia Agricultural University, Hohhot 010018, China; 15147035388@163.com (M.Z.); hamuqier1989@126.com (Q.M.); liuna_dky@163.com (N.L.); shuaimaomn147@126.com (Y.S.); 2 Key Laboratory of Smart Animal Husbandry at Universities of Inner Mongolia Autonomous Region, and Research Center of Dairy Cattle Breeding and Farming Technology, National Center of Technology Innovation for Dairy Industry, Inner Mongolia Agricultural University, Hohhot 010018, China

**Keywords:** *Glycyrrhiza uralensis* Fisch. stems, enzymatic hydrolysis, high-temperature steam treatment, cell wall remodeling, flavonoid metabolism, antioxidant

## Abstract

*Glycyrrhiza uralensis* Fisch. (*G. uralensis*) stems are an underutilized agricultural by-product rich in polysaccharides, phenolics, and flavonoids. This study systematically compared enzymatic and high-temperature steam treatments for improving their functional value. Both treatments altered plant matrix architecture but exhibited distinct effects. Enzymatic processing largely preserved the original color and induced moderate cell wall loosening, whereas steam treatment caused more extensive tissue disruption, porous structure formation, and thermal browning. Enzymatic treatment increased extract solubility from 0.25% to 0.32%, soluble polysaccharide content from 266.06 ± 1.58 to 319.40 ± 4.19 mg/g, and total phenolic content from 49.69 ± 0.65 to 63.99 ± 0.81 mg/g. In contrast, steam treatment reduced solubility to 0.14% and total phenolic content to 38.39 ± 0.52 mg/g, despite increasing polysaccharide content to 300.50 ± 3.02 mg/g. Metabolomic analysis revealed divergent flavonoid remodeling between the two processing strategies, with enzymatic treatment associated with distinct changes in the relative abundance of several flavonoid subclasses and metabolites. Consistently, enzyme-treated extracts showed superior DPPH radical-scavenging activity (90.90% at 16 mg/mL) and markedly improved embryo survival and hatching rates (91.25%) in an AAPH-induced zebrafish model. Collectively, these findings highlight that controlled enzymatic remodeling, rather than excessive thermal disruption, is more effective for preserving functional molecules and enhancing the bioactive potential of *G. uralensis* stems.

## 1. Introduction

*Glycyrrhiza uralensis* Fisch. (*G. uralensis*) is an important medicinal and edible plant widely used in traditional medicine [1] due to its abundant bioactive constituents, including flavonoids, triterpenoid saponins, and polysaccharides [2]. Although the roots of *G. uralensis* have been extensively investigated and utilized, the aerial parts [3], particularly stems, are generally underexplored and often discarded or used for low-value applications during processing, resulting in considerable resource waste [4]. Recent studies have demonstrated that *G. uralensis* stems contain diverse phenolic compounds, flavonoids, and polysaccharides with potential antioxidant and functional food values [5]. Therefore, developing effective processing strategies to enhance the accessibility and utilization of bioactive components in *G. uralensis* stems is essential for promoting their high-value exploitation.

Thermal processing and enzymatic modification represent two major strategies for improving the functional properties of plant-derived materials. Conventional thermal treatments can facilitate tissue disruption and promote the release of certain bioactive compounds; however, excessive heating may also induce oxidation of phenolics, cleavage of glycosidic bonds, and degradation of heat-sensitive metabolites, thereby compromising functional quality [6,7]. In contrast, enzymatic processing provides a mild and selective approach by degrading structural polysaccharides, such as pectin, cellulose, and hemicellulose, to enhance the release of bound bioactive compounds while minimizing thermal damage [8,9]. Although enzymatic and steam-based processing have individually been investigated in plant-derived materials, a direct comparison of pectinase-assisted and high-temperature steam treatments in *G. uralensis* stems under defined processing conditions remains lacking. In particular, it remains unclear whether these two strategies induce distinct structural remodeling and associated changes in chemical composition, flavonoid profiles, and antioxidant-related phenotypes. In this study, pectinase was selected as the enzymatic strategy because it provides a relatively mild and targeted approach to modifying pectin-containing regions of plant cell walls, potentially improving matrix accessibility while minimizing the thermal damage associated with conventional heat processing [9,10,11]. Our previous studies have demonstrated the applicability of pectinase-assisted processing [12] and high-temperature steam treatment [13] to *G. uralensis* Fisch, respectively, providing a methodological basis for comparing these two processing strategies in *G. uralensis* stems.

Consequently, this study systematically compared the effects of enzymatic and high-temperature steam treatments on the physicochemical characteristics, structural properties, chemical composition, metabolic profiles, and antioxidant activities of *G. uralensis* stems. By integrating microscopic structural characterization, chemical composition analysis, untargeted metabolomics, *in vitro* antioxidant assays, and an oxidative stress zebrafish model, we aimed to elucidate the differential mechanisms underlying processing-induced bioactive compound transformation. This study provides new insights into how processing strategies regulate functional ingredient accessibility in *G. uralensis* stems and offers a theoretical basis for their high-value utilization and development as functional food resources.

## 2. Materials and Methods

### 2.1. Sample Collection and Pretreatment

The stems of *G. uralensis* were collected from cultivated fields located in Hangjin Banner, Ordos City, Inner Mongolia Autonomous Region, China (39°36′ N, 109°47′ E). After harvesting, impurities including senescent leaves, weeds, and petioles were carefully removed, and only young stem segments were retained as the experimental materials. The collected stems were thoroughly mixed to ensure sample homogeneity and subsequently used as the raw material for further analysis. The samples were naturally air-dried, pulverized into powder, and passed through a 60-mesh sieve (250–380 μm). The resulting powdered material was used as the experimental substrate for all subsequent enzymatic and high-temperature steam treatments. Detailed information regarding the major reagents, instruments, and equipment used in this study is provided in Table S1. Unless otherwise specified, all chemicals were of analytical grade.

### 2.2. Preparation of Processed G. uralensis Stem Samples

Sample preparation was performed according to our previously reported method with minor modifications. Three types of samples were prepared, including enzyme-treated *G. uralensis* stem (EGS), high-temperature steam-treated *G. uralensis* stem (HGS), and untreated *G. uralensis* stems (GS). For enzymatic treatment [14], the powdered *G. uralensis* stems were mixed with distilled water at a solid-to-liquid ratio of 1:0.7 (*w*/*v*), followed by the addition of a commercial pectinase preparation (P8182, Beijing Solarbio Science & Technology Co., Ltd., Beijing, China; enzymatic activity: 100,000 U/g, according to the manufacturer’s specification) at 1.2% (w/w, based on dry weight). No pH adjustment or buffer was applied during enzymatic hydrolysis. The mixture was thoroughly homogenized and transferred into multilayer polyethylene fermentation bags (20 × 18 cm) equipped with one-way gas release valves. After removing excess air, the bags were sealed and subjected to static enzymatic hydrolysis at 50 °C for 36 h in a temperature-controlled incubator. The treated samples were subsequently dried at 60 °C to constant weight to obtain EGS samples. For high-temperature steam treatment [13], dried *G. uralensis* stems were processed in an autoclave at 120 °C under 103 kPa for 10 min, followed by air-drying at room temperature to obtain HGS samples. Untreated dried *G. uralensis* stems without additional processing were used as controls (GS).

The dried GS, EGS, and HGS samples were pulverized and passed through a 60-mesh sieve (250–380 μm). The obtained powders were subjected to subsequent analyses of physicochemical properties and structural characteristics. For the preparation of water extracts, GS, EGS, and HGS powders were individually extracted with distilled water at a solid-to-liquid ratio of 1:16 (w/v) at 80 °C for 30 min under shaking conditions (every 5 min). After cooling, the extracts were centrifuged at 4000 rpm for 15 min, and the supernatants were collected and concentrated using a rotary evaporator. The resulting extracts were designated as GS extract (GSE), EGSE, and HGSE, respectively, and were used for subsequent analyses of chemical composition and antioxidant activities.

### 2.3. Quantitative Color Analysis of Processed G. uralensis Stem Samples

The color characteristics were initially evaluated utilizing a custom-built integrated image acquisition system. The system consisted of a D65 standard light cabinet (color temperature: 6500 K) [15], a Hikvision industrial camera, and a sample platform. Specifically, 3 g of samples were evenly distributed in Petri dishes with a diameter of 6 cm. The sample surface was carefully leveled to minimize particle accumulation, uneven distribution, and shadow effects. Then, each dish was placed at a fixed position on the sample platform under identical illumination conditions for image capture. The camera parameters were set as follows: resolution of 2592 × 1944 pixels, aperture of f/8; shutter speed of 1/60 s; ISO 200; and white balance fixed to the D65 standard. Five images were acquired for each sample under identical conditions, each representing an independent sampling region. The acquired RGB images were converted into 8-bit grayscale images, and the mean gray value was calculated. Subsequently, the average values of the red (R), green (G), and blue (B) channels were extracted, and the RGB values were further transformed into the CIELAB color space to obtain lightness (L*), redness (a*), and yellowness (b*).

### 2.4. Physicochemical Properties and Structural Characterization

The physicochemical properties and structural characteristics were determined according to our previously reported methods [16]. Briefly, water-holding capacity (WHC) was determined by hydration and centrifugation of samples, while solubility (S) and swelling power (SP) were measured after heating the samples in water followed by centrifugation and drying of the supernatant. The microstructural characteristics were examined applying scanning electron microscopy (SEM). Dried powders were mounted on conductive adhesive tape, sputter-coated with gold, and observed at 1000× magnification. SEM images were further analyzed through ImageJ software (v 1.4) based on gray-level co-occurrence matrix (GLCM) analysis, including contrast, correlation, energy, and inverse difference moment (IDM). Furthermore, thermogravimetric analysis (TGA) was conducted under a nitrogen atmosphere (40 mL/min) at a heating rate of 10 °C/min from 50 to 800 °C. X-ray diffraction (XRD) patterns were recorded with Cu Kα radiation (λ = 0.15406 nm) over a scanning range of 2θ = 5–40°.

### 2.5. Determination of Chemical Compositions and Polysaccharide Characterization

Also, the contents of polysaccharides and total phenolic compounds were determined following our previously reported methods, namely the phenol–sulfuric acid method and Folin–Ciocalteu method, respectively [17,18]. Briefly, glucose served as the standard for polysaccharide quantification; absorbance was measured at 490 nm, and polysaccharide content was calculated using the corresponding calibration curve. For total phenolics, gallic acid was used as the standard. After reaction with Folin–Ciocalteu reagent and sodium carbonate solution, absorbance was measured at 760 nm to calculate total phenolic content according to the gallic acid calibration curve. The monosaccharide composition of polysaccharides was analyzed using 1-phenyl-3-methyl-5-pyrazolone (PMP) pre-column derivatization coupled with high-performance liquid chromatography (HPLC) [19]. Expressly, polysaccharide samples were hydrolyzed, derivatized with PMP, and analyzed using an Agilent 1200 HPLC system (Agilent Technologies, Santa Clara, CA, USA) equipped with a C18 column. Individual monosaccharides were identified and quantified by comparison with corresponding monosaccharide standards. The structural characteristics of polysaccharides were further analyzed by Fourier-transform infrared spectroscopy (FT-IR) [20]. Dried polysaccharide samples were mixed with KBr, compressed into pellets, and scanned over the range of 4000–500 cm^−1^.

### 2.6. Untargeted Metabolomics Profiling and Bioinformatics Analysis

Untargeted metabolomic profiling was performed through ultra-performance liquid chromatography coupled with tandem mass spectrometry (UPLC–MS/MS) according to a previously reported method with minor modifications [16]. Primarily, 50 mg of sample powder was extracted with 1200 μL of pre-cooled 70% methanol containing L-2-chlorophenylalanine as an internal standard (0.02 mg/mL). After repeated vortexing and centrifugation at 4 °C, the supernatants were filtered through a 0.22 μm membrane. Chromatographic separation was performed using an ExionLC^TM^ AD system (SCIEX, Framingham, MA, USA) equipped with an Agilent SB-C18 column (Agilent Technologies, Santa Clara, CA, USA), and mass spectrometric detection was conducted on a QTRAP 6500+ system equipped with an electrospray ionization source under both positive and negative ion modes. Metabolite identification was achieved by matching MS/MS spectra against the MetWare database (https://www.metware.cn/mwSjk.html?questionCateId=3; accessed on 1 March 2026). Quantification was performed operating MultiQuant software (v 3.0.3) based on integrated multiple reaction monitoring (MRM) peak areas normalized to internal standards. Multivariate statistical analyses, including principal component analysis (PCA) and orthogonal partial least squares discriminant analysis (OPLS-DA), were performed using SIMCA software (v 14.1). Differentially expressed metabolites (DEMs) were identified according to the criteria of variable importance in projection (VIP) > 1, *p* < 0.05, and |log_2_fold change (FC)| ≥ 1. Pearson correlation analysis was conducted to evaluate associations between DEMs and antioxidant-related parameters. Kyoto encyclopedia of genes and genomes (KEGG) analysis was performed making use of legume species as the reference background, with *p* < 0.05 considered statistically significant.

### 2.7. Antioxidant Activity Assessment of G. uralensis Stem Extracts

#### 2.7.1. *In Vitro* Antioxidant Assays

The *in vitro* antioxidant activities of GSE, EGSE, and HGSE were evaluated by 1,1-diphenyl-2-picrylhydrazyl (DPPH) radical scavenging activity, hydroxyl radical scavenging activity, and total reducing power assays according to previously established methods [17]. Five concentrations of each extract (0, 2, 4, 8, and 16 mg/mL) were evaluated. Moreover, to further quantify antioxidant efficacy, the half-maximal inhibitory concentration (IC_50_) values were calculated for each sample.

#### 2.7.2. Zebrafish Oxidative Stress Model and Antioxidant Evaluation

Wild-type AB zebrafish (4–5 months old) were obtained from the China Zebrafish Resource Center and maintained in a recirculating aquatic system at 28.5 ± 1 °C under a 14 h light/10 h dark photoperiod. Adult fish were fed daily with formulated feed and freshly hatched Artemia nauplii. Embryos were obtained by natural spawning and collected within 30 min after fertilization. Normally developed and transparent embryos were selected under a stereomicroscope for subsequent experiments. GSE, EGSE, and HGSE were dissolved in embryo medium to prepare working suspensions. Based on previous studies from our group [12,13], 50 μg/mL was selected as the safe concentration for treatment. Embryos were randomly distributed into 24-well plates (10 embryos/well, 2 mL medium per well), with four biological replicates per group.

To establish the oxidative stress model, embryos were divided into five groups: the negative control group (NC, embryo medium only), 2,2′-Azobis(2-amidinopropane) dihydrochloride (AAPH) model group (15 mmol/L AAPH), GSE group (50 μg/mL GSE + 15 mmol/L AAPH), EGSE group (50 μg/mL EGSE + 15 mmol/L AAPH), and HGSE group (50 μg/mL HGSE + 15 mmol/L AAPH). Specifically, at 7–9 h post-fertilization (hpf), embryos were pretreated with corresponding extracts for 1 h, followed by exposure to AAPH (final concentration: 15 mmol/L). The culture medium was renewed every 12 h, and embryos were maintained at 28 °C until 60 hpf. Moreover, at 60 hpf, embryo survival and hatching rates were recorded under a stereomicroscope. Mortality was defined based on the absence of heartbeat, spontaneous movement, or embryo pigmentation abnormalities. For morphological evaluation, 10 randomly selected larvae from each group were anesthetized with MS-222 (0.02%) and imaged laterally. Body length and yolk sac area were quantified using ImageJ software (v 1.4). The relative yolk sac area was normalized to the mean value of the NC group (100%).

### 2.8. Statistical Analysis

All experiments were performed with three independent biological replicates, and each sample was analyzed in triplicate unless otherwise stated. Data are presented as mean ± standard deviation (SD). Additionally, statistical analyses were performed utilizing IBM SPSS (v 26.0). For comparisons among multiple groups, one-way analysis of variance (ANOVA) followed by Duncan’s multiple range test was applied. The assumptions of normality and homogeneity of variance were evaluated using the Shapiro–Wilk test and Levene’s test, respectively, prior to ANOVA. Statistical significance was defined as *p* < 0.05. All figures were generated by GraphPad Prism (v 10.1.2).

## 3. Results

### 3.1. High-Temperature Steam Treatment Markedly Darkens G. uralensis Stems Compared with Enzymatic Treatment

**Figure 1:**
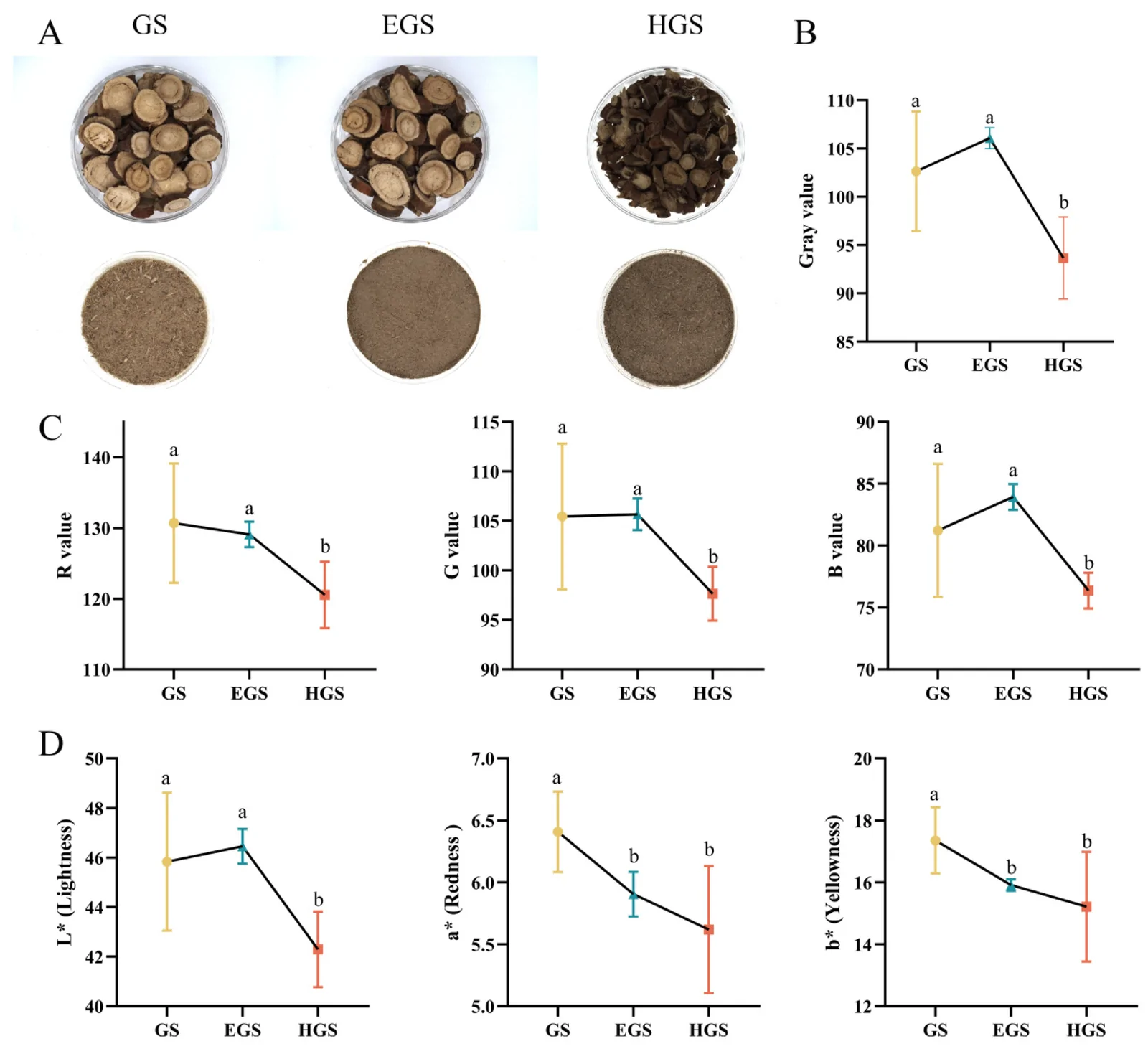
Processing-induced alterations in the color characteristics of *G. uralensis* stems. (**A**) Representative images of sliced sections (upper panel) and powdered samples (lower panel) of untreated *G. uralensis* stems (GS), enzyme-treated *G. uralensis* stems (EGS), and high-temperature steam-treated *G. uralensis* stems (HGS). (**B**) Gray value. (**C**) Red (R), green (G), and blue (B) values. (**D**) CIELAB color parameters, including lightness (L*), redness (a*), and yellowness (b*). Different lowercase letters denote significant differences between groups (*p* < 0.05), whereas identical letters indicate no significant difference (*p* > 0.05).

Color transformation represents one of the most apparent phenotypic changes during processing and directly influences the sensory characteristics of *G. uralensis* products. Visually, GS sections exhibited a natural pale yellowish-white appearance with clear and bright fibrous structures. EGS sections showcased slight browning with a relatively uniform and softer color appearance, whereas HGS sections displayed extensive dark brown coloration, indicating pronounced browning after high-temperature steam treatment. Similar trends were observed in powdered samples: GS exhibited a light yellow-brown color with visible fibrous fragments, EGS showed a moderately darkened but homogeneous brown appearance, while HGS exhibited obvious darkening and severe browning (Figure 1A). Quantitative color analysis further confirmed these visual differences. The gray value of EGS showed no significant difference compared with GS (*p* > 0.05), suggesting that enzymatic treatment had limited effects on overall brightness. In contrast, HGS exhibited a significantly decreased gray value compared with GS (*p* < 0.05), indicating substantial darkening induced by high-temperature steam processing (Figure 1B). Consistently, the R, G, and B channel values of EGS were comparable to those of GS (*p* > 0.05), whereas all three parameters were significantly reduced in HGS (*p* < 0.05), accounting for its darker brown appearance (Figure 1C). The CIELAB parameters further characterized processing-associated color variations. The L* value of HGS was significantly lower than those of GS and EGS (*p* < 0.05), consistent with the grayscale results. The a* value was highest in GS and significantly decreased after both enzymatic and high-temperature steam treatments (*p* < 0.05), indicating reduced redness intensity. Similarly, the b* value followed the trend of GS > EGS ≈ HGS, with GS exhibiting significantly higher yellowness compared with processed samples (*p* < 0.05) (Figure 1D).

### 3.2. Enzymatic and High-Temperature Steam Treatment Induce Distinct Microstructural Remodeling of G. uralensis Stems

**Figure 2:**
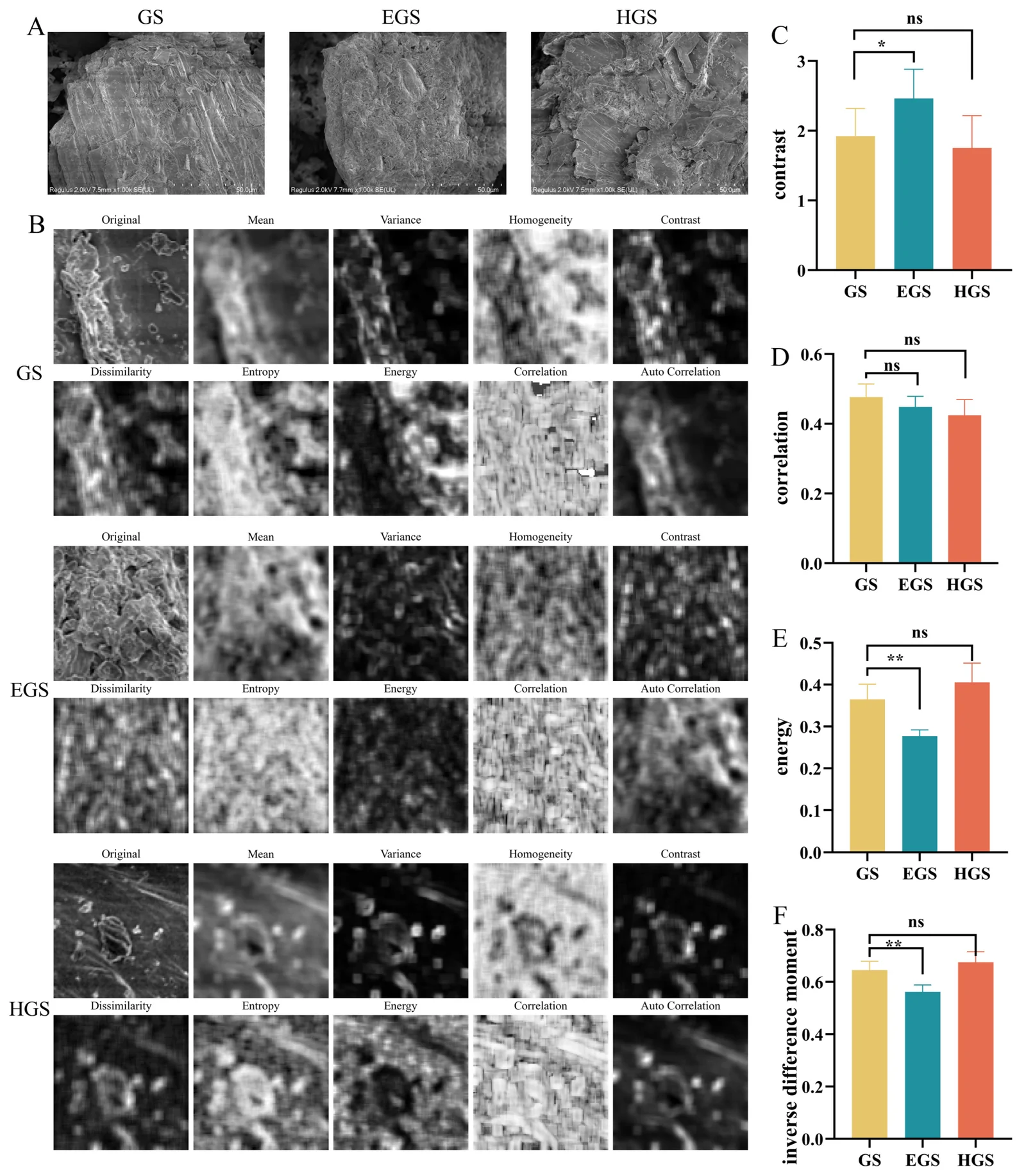
Processing-induced alterations in the microstructure and surface texture characteristics of *G. uralensis* stems. (**A**) Representative scanning electron microscopy (SEM) images of untreated *G. uralensis* stems (GS), enzyme-treated *G. uralensis* stems (EGS), and high-temperature steam-treated *G. uralensis* stems (HGS) at 1000× magnification. (**B**) Representative gray-level co-occurrence matrix (GLCM)-derived texture features of GS, EGS, and HGS samples. (**C**) Contrast. (**D**) Correlation. (**E**) Energy. (**F**) Inverse difference moment (IDM). * *p* < 0.05, ** *p* < 0.01 and ns: *p* > 0.05.

Processing-induced changes were further characterized at the microstructural level by SEM. GS exhibited relatively intact and smooth surfaces with well-organized fibrous structures and clearly defined cell wall boundaries. In contrast, EGS showed apparent surface erosion and partial structural disruption, accompanied by loosened fiber bundles and the formation of irregular pores and wrinkles, resulting in a more porous and sponge-like morphology. HGS displayed a distinct structural pattern characterized by severe surface collapse, fractured cell walls, and abundant irregular fragments and pores, indicating more extensive cell wall disruption caused by high-temperature steam treatment (Figure 2A). To quantitatively evaluate the surface structural variations, representative SEM regions (200 × 200 pixels) were subjected to GLCM analysis, including contrast, correlation, energy, and IDM (Figure 2B). The contrast value was significantly increased in EGS compared with GS (*p* < 0.05), whereas no significant difference was observed between HGS and GS (*p* > 0.05), suggesting enhanced local surface heterogeneity after enzymatic treatment (Figure 2C). Notably, the correlation parameter showed no significant differences among the three groups (*p* > 0.05) (Figure 2D). Similarly, energy and IDM exhibited comparable trends, with both parameters significantly decreased in EGS compared with GS (*p* < 0.01), while HGS showed no significant difference from GS (*p* > 0.05) (Figure 2E,F). In summary, EGS showed localized surface disruption and increased surface heterogeneity, while HGS exhibited more extensive structural disruption.

### 3.3. Enzymatic and High-Temperature Steam Treatment Differentially Remodel Hydration Properties, Crystalline Structure, and Thermal Stability of G. uralensis Stems

**Figure 3:**
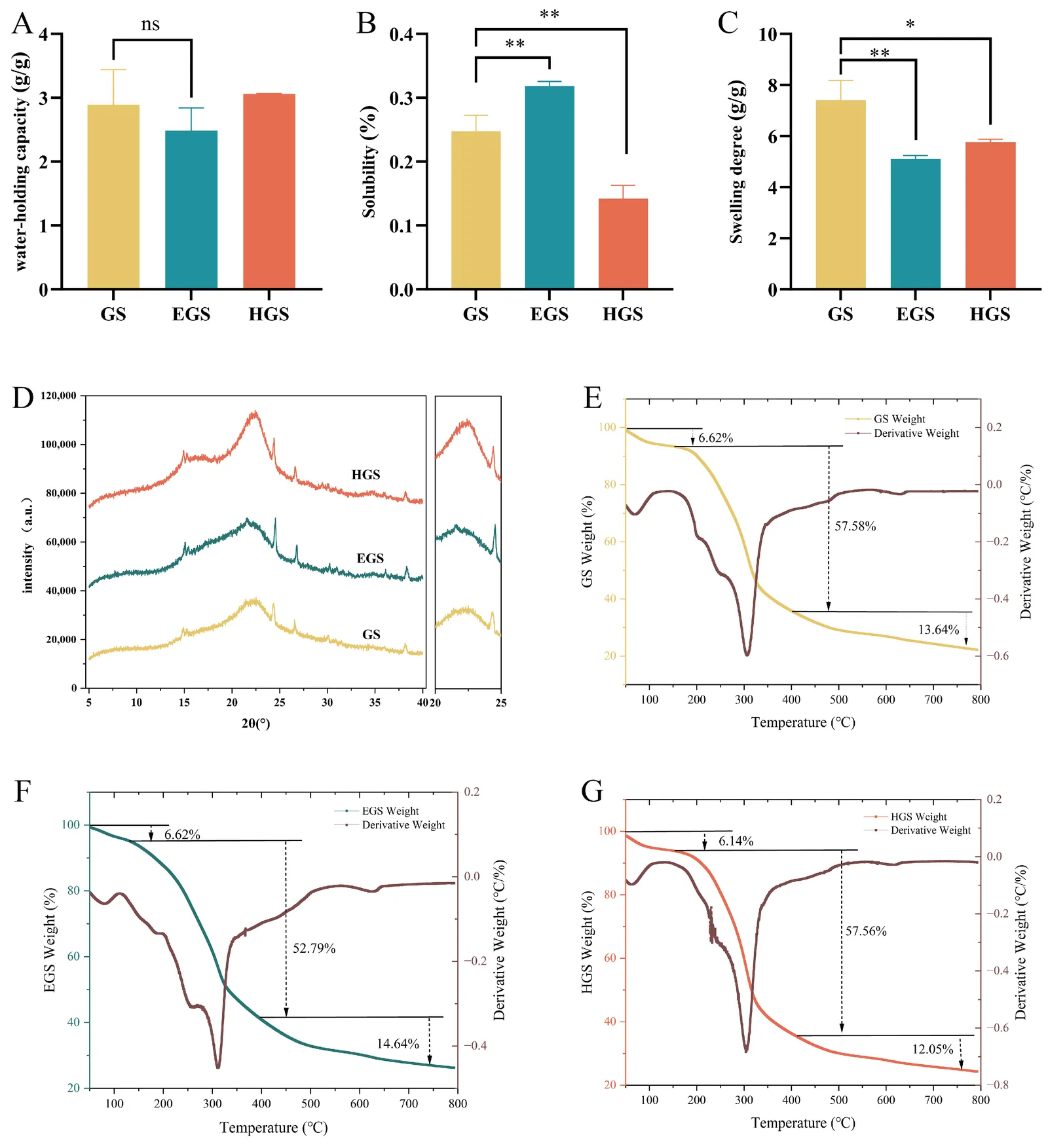
Processing-induced alterations in hydration properties, crystalline structure, and thermal stability of *G. uralensis* stems. (**A**) Water-holding capacity (WHC). (**B**) Solubility (S). (**C**) Swelling power (SP). (**D**) X-ray diffraction (XRD) patterns. (**E**–**G**) Thermogravimetric analysis (TGA) and derivative thermogravimetric (DTG) curves of untreated *G. uralensis* stems (GS), enzyme-treated *G. uralensis* stems (EGS), and high-temperature steam-treated *G. uralensis* stems (HGS). * *p* < 0.05, ** *p* < 0.01, ns = not significant.

The effects of different processing strategies on the physicochemical characteristics of *G. uralensis* stems were further evaluated. The WHC of GS, EGS, and HGS remained within a narrow range (2.49–3.06 g/g), indicating that neither enzymatic nor high-temperature steam treatment substantially disrupted the intrinsic water-binding network (Figure 3A). In contrast, EGS exhibited significantly increased S compared with GS (0.32% vs. 0.25%, *p* < 0.05), whereas HGS showed a marked decrease (0.14%, *p* < 0.05) (Figure 3B). The SP displayed an opposite trend, decreasing from 7.41 g/g in GS to 5.10 and 5.77 g/g in EGS and HGS, respectively (*p* < 0.05) (Figure 3C). XRD revealed that all samples retained characteristic cellulose diffraction patterns within the 2θ range of approximately 15–25°, indicating that neither treatment completely disrupted the cellulose crystalline framework. However, processing altered diffraction intensity and peak morphology. The intensity of the cellulose-related diffraction peaks (15°) increased progressively from GS to EGS and HGS, suggesting altered cellulose crystallinity after thermal processing. Meanwhile, EGS exhibited a slight shift in the (002) diffraction peak toward lower angles and reduced peak sharpness, indicating partial lattice expansion or increased structural disorder after enzymatic treatment. In contrast, HGS displayed sharper diffraction peaks, suggesting a relatively higher degree of crystalline ordering after thermal processing (Figure 3D).

TGA and derivative thermogravimetric (DTG) curves further demonstrated processing-dependent differences in thermal degradation behavior. All samples exhibited similar initial weight loss (6.14–6.62%, 30–150 °C) associated with moisture evaporation, consistent with their comparable WHC. The second stage (150–400 °C) corresponded to the main decomposition process, with weight losses of 52.79% (GS), 57.56% (EGS), and 57.58% (HGS). The third stage (400–800 °C) was associated with the further thermal degradation of residual material, showing weight losses in the order EGS (14.64%) > GS (13.64%) > HGS (12.05%). At 800 °C, the char yields followed EGS (26.24%) > HGS (24.27%) > GS (22.20%). The DTG profiles revealed distinct degradation patterns: HGS exhibited the sharpest degradation peak (303 °C, −0.68%/°C) and an additional low-temperature shoulder peak (230 °C, −0.26%/°C), suggesting more heterogeneous thermal decomposition, whereas EGS displayed a broader and lower degradation peak (311 °C, −0.45%/°C), indicating modified degradation kinetics (Figure 3E–G). Overall, enzymatic and high-temperature steam treatments induced distinct physicochemical and structural changes in *G. uralensis* stems. Enzymatic treatment was associated with increased solubility and moderate structural remodeling, whereas high-temperature steam treatment resulted in greater changes in crystalline structure and thermal degradation behavior.

### 3.4. Processing-Dependent Enhancement and Remodeling of Bioactive Components in G. uralensis Stem Extracts

**Figure 4:**
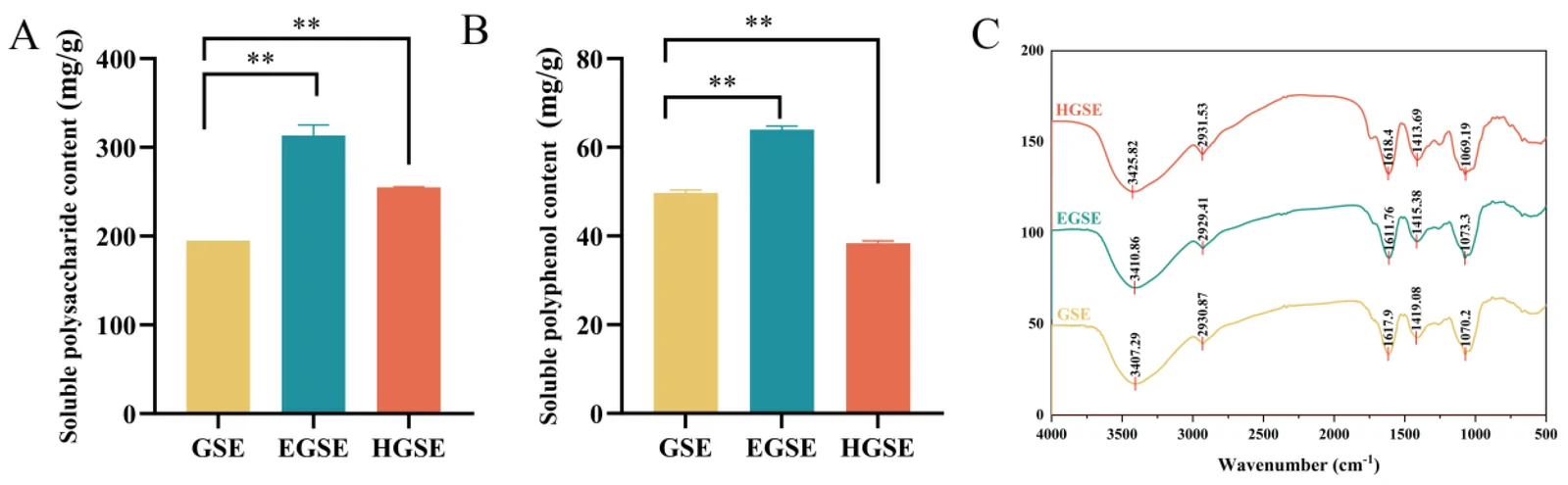
Processing-induced changes in bioactive components and functional groups of *G. uralensis* stems extracts. (**A**) Soluble polysaccharide content. (**B**) Total phenolic content. (**C**) Fourier-transform infrared (FT-IR) spectra of untreated *G. uralensis* stems extract (GSE), enzyme-treated *G. uralensis* stems extract (EGSE), and high-temperature steam-treated *G. uralensis* stems extract (HGSE). ** *p* < 0.01.

The polysaccharide content of GSE was 266.06 ± 1.58 mg/g, which significantly increased to 319.40 ± 4.19 mg/g in EGSE and 300.50 ± 3.02 mg/g in HGSE (*p* < 0.01) (Figure 4A). In contrast, total phenolic content exhibited a distinct processing-dependent pattern. EGSE showed a significant increase compared with GSE (63.99 ± 0.81 vs. 49.69 ± 0.65 mg/g, *p* < 0.01), whereas HGSE displayed a marked reduction (38.39 ± 0.52 mg/g, *p* < 0.01) (Figure 4B). Monosaccharide profiling further revealed processing-induced remodeling of polysaccharide composition. Glucose was the predominant monosaccharide in GSE (76.64 mol%), followed by galactose (5.82 mol%), arabinose (4.68 mol%), and galacturonic acid (3.49 mol%). After enzymatic treatment, the proportion of glucose slightly decreased to 71.07 mol%, accompanied by increased galacturonic acid (5.87 mol%) and rhamnose (2.07 mol%) contents, with trace amounts of mannuronic acid detected. High-temperature steam treatment induced more pronounced compositional alterations, characterized by a substantial decrease in glucose proportion (46.64 mol%) and marked increases in arabinose (15.53 mol%) and galacturonic acid (20.58 mol%). Several monosaccharides, including guluronic acid and N-acetylgalactosamine, were no longer detected in HGSE (Table 1). FT-IR analysis further confirmed the preservation of the major functional groups of polysaccharides after processing. The broad O–H stretching bands around 3400 cm^−1^ [21] and C–H stretching bands near 2930 cm^−1^ [22,23] showed minimal differences among the three groups. The absorption band around 1610–1620 cm^−1^, assigned to carbonyl-related vibrations [24], exhibited a slight shift after enzymatic treatment and decreased intensity after high-temperature treatment, consistent with the changes in phenolic content. Peaks around 1415 cm^−1^ and 1070 cm^−1^ corresponding to C–H bending [25] and C–O–C/C–OH [17] stretching vibrations remained largely unchanged, indicating that processing did not substantially alter the basic polysaccharide backbone structure (Figure 4C). Collectively, enzymatic treatment was associated with increased soluble polysaccharide and phenolic contents, together with relatively limited changes in the overall polysaccharide spectral features. High-temperature steam treatment, in contrast, was associated with more pronounced changes in monosaccharide composition and reduced phenolic content.

### 3.5. Processing-Dependent Metabolic Remodeling Reveals Flavonoid-Rich DEMs in G. uralensis Stems Extracts

**Figure 5:**
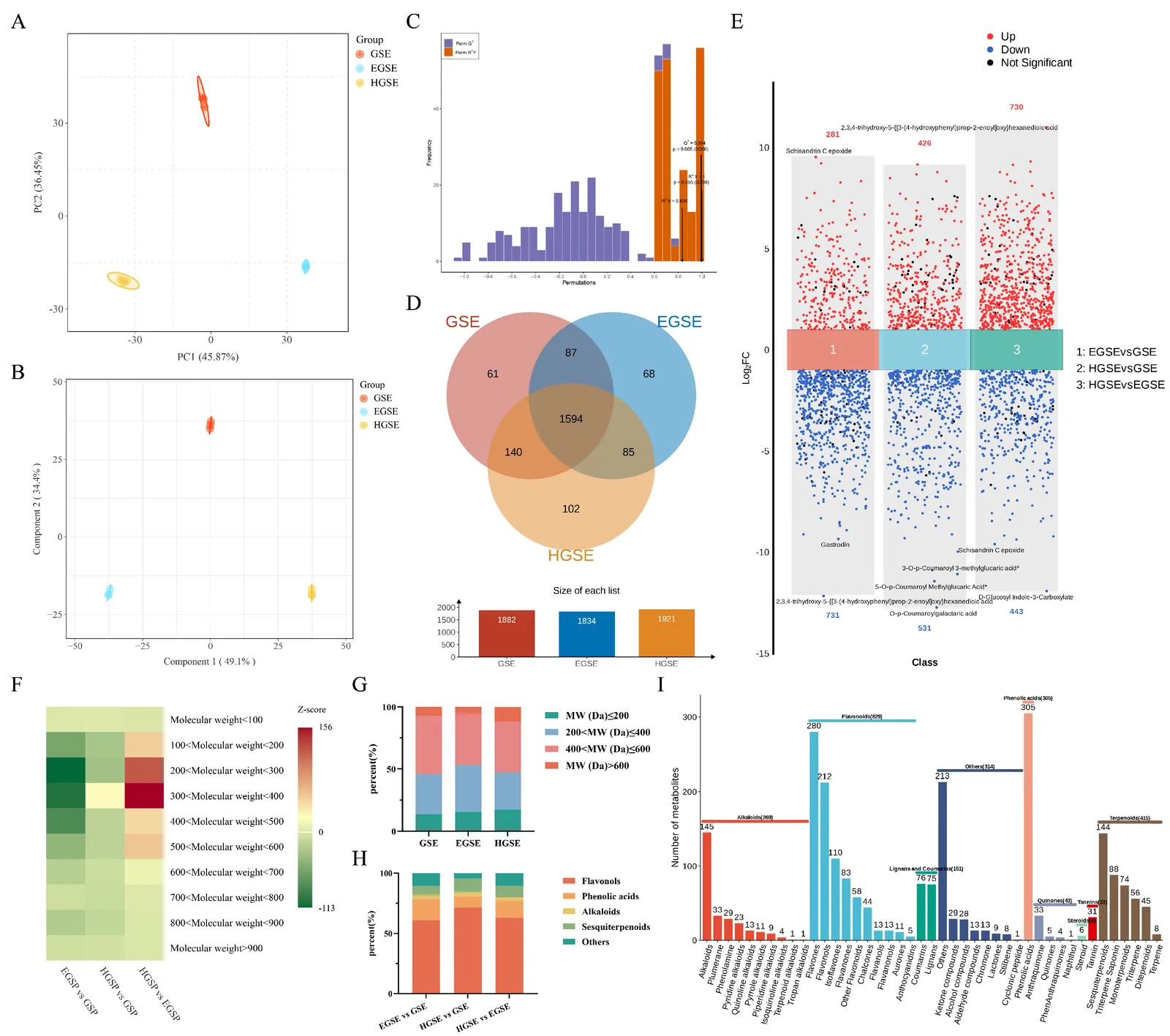
Untargeted metabolomic profiling reveals processing-dependent metabolic signatures and differentially expressed metabolites (DEMs) characteristics in *G. uralensis* stem extracts. (**A**) Principal component analysis (PCA) score plot. (**B**) Orthogonal partial least squares discriminant analysis (OPLS-DA) score plots. (**C**) Permutation tests of OPLS-DA models. (**D**) Venn diagram showing shared metabolites among untreated *G. uralensis* stems extract (GSE), enzyme-treated *G. uralensis* stems extract (EGSE), and high-temperature steam-treated *G. uralensis* stems extract (HGSE). (**E**) Volcano plot showing DEMs among different processing comparisons. (**F**) Heatmap of DEMs abundance distribution across different molecular weight ranges. (**G**) Stacked bar plot showing the proportion of DEMs within different molecular weight intervals. (**H**) Superclass distribution of DEMs within the 200–600 Da molecular weight range. (**I**) Classification distribution of DEMs at superclass and subclass levels.

Untargeted metabolomics was subsequently performed to characterize processing-induced metabolic alterations among GSE, EGSE, and HGSE samples. PCA revealed clear separation among the three groups, with PC1 and PC2 explaining 82.32% of the total variance (Figure 5A). OPLS-DA further demonstrated complete discrimination among pairwise comparisons of the three processing groups (Figure 5B). Permutation tests (200 iterations) showed high model reliability (R^2^X = 0.835, R^2^Y = 1.000, Q^2^ = 0.994), indicating no apparent overfitting (Figure 5C). A total of 1882, 1834, and 1921 metabolites were detected in GSE, EGSE, and HGSE, respectively, with 1594 metabolites shared among all three groups (Figure 5D). Differential metabolite analysis based on VIP > 1, *p* < 0.05, and |log_2_FC| ≥ 1 identified 1012 DEMs between EGSE and GSE, including 281 upregulated and 731 downregulated metabolites. Comparison between HGSE and GSE revealed 957 DEMs (426 upregulated and 531 downregulated), whereas 1173 DEMs were identified between EGSE and HGSE (730 upregulated and 443 downregulated) (Figure 5E). Molecular weight distribution analysis showed that most DEMs were concentrated within the 200–600 Da range, whereas metabolites with molecular weights below 100 Da or above 600 Da accounted for a relatively small proportion (Figure 5F). Consistently, metabolites within the 200–400 Da and 400–600 Da ranges represented more than 80% of total DEMs in all three groups (Figure 5G). Among the highly abundant metabolites within this range, flavonoids represented the predominant superclass, followed by phenolic acids, with comparable overall chemical class distributions among the three samples (Figure 5H). Further subclass classification revealed distinct processing-associated metabolic patterns. Enzymatic treatment markedly increased multiple flavonoid subclasses, including flavonol glycosides, dihydroflavonoid glycosides, and isoflavone glycosides. In contrast, high-temperature steam treatment resulted in decreased levels of modified flavonoids, particularly malonylated and acylated flavonoid glycosides. Several terpenoid saponins and phenylpropanoid metabolites were moderately increased after enzymatic treatment, although their changes were less pronounced than those of flavonoid compounds. Given their relatively high representation among the differentially affected metabolites and their pronounced responses to processing (Figure 5I), flavonoid-related metabolites were selected for further comparative analysis and correlation with antioxidant-related parameters.

### 3.6. Enzymatic and High-Temperature Steam Treatment Induce Divergent Remodeling of Flavonoid Metabolic Profiles

**Figure 6:**
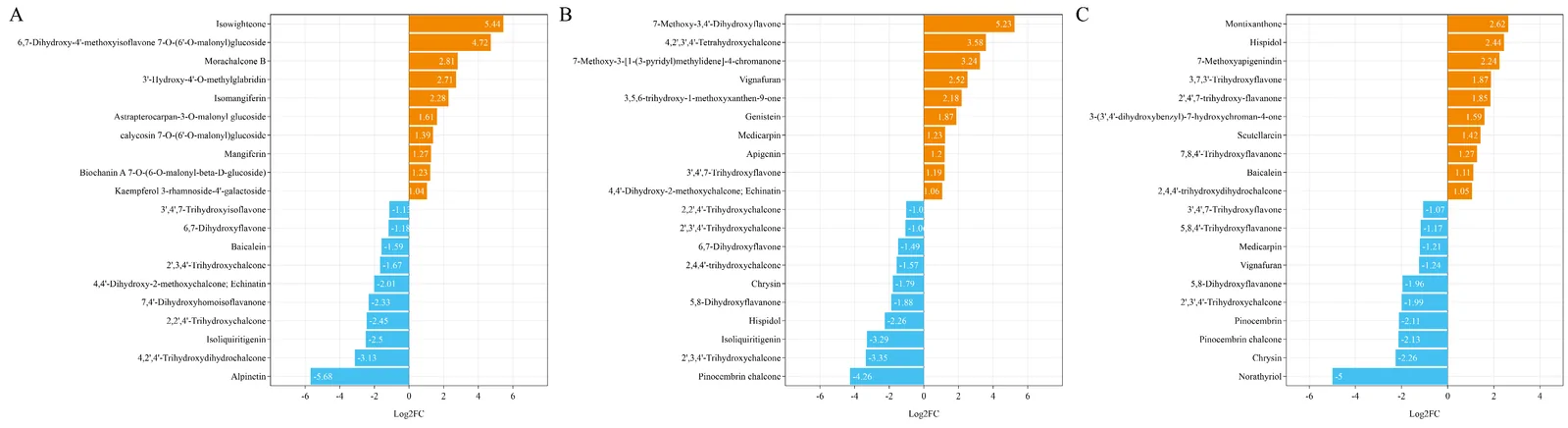
Differential regulation patterns of flavonoid metabolites within the 200–600 Da molecular weight range among different processing comparisons. (**A**) Top 10 upregulated and downregulated flavonoid differential metabolites in enzyme-treated *G. uralensis* stems extract (EGSE) vs. untreated *G. uralensis* stems extract (GSE) comparison. (**B**) Top 10 upregulated and downregulated flavonoid differential metabolites in high-temperature steam-treated *G. uralensis* stems extract (HGSE) vs. GSE comparison. (**C**) Top 10 upregulated and downregulated flavonoid differential metabolites in the EGSE vs. HGSE comparison. Orange indicates upregulated metabolites (log_2_FC > 0), and blue indicates downregulated metabolites (log_2_FC < 0).

Since flavonoids represented the predominant class among the identified differential metabolites, flavonoid compounds within the 200–600 Da molecular weight range were further characterized based on their log_2_FC to identify processing-responsive metabolites. Compared with GSE, enzymatic treatment induced substantial alterations in flavonoid accumulation patterns in EGSE samples. Several flavonoid glycosides and related derivatives exhibited increased relative abundance, whereas multiple chalcones, flavonoid aglycones, and hydroxylated flavonoids showed decreased levels (Figure 6A). High-temperature steam treatment also resulted in pronounced flavonoid remodeling compared with GSE. The altered metabolites included both increased and decreased flavonoid subclasses, suggesting that thermal processing induced complex transformation of flavonoid components rather than a uniform enrichment or depletion pattern (Figure 6B). Direct comparison of EGSE versus HGSE further highlighted the divergence between the two processing regimes. High-temperature steam treatment was enriched in montixanthone, hispidol and 7-methoxyapigeninidin, whereas enzymatic extracts retained higher levels of norathyriol, chrysin and pinocembrin chalcone (Figure 6C). These findings indicate that enzymatic and high-temperature steam treatments generated distinct flavonoid metabolite patterns, although the absolute concentrations and individual biological contributions of these metabolites require further targeted validation.

### 3.7. Enzymatic Processing Enhances In Vitro Antioxidant Capacity of G. uralensis Stems Extracts

**Figure 7:**
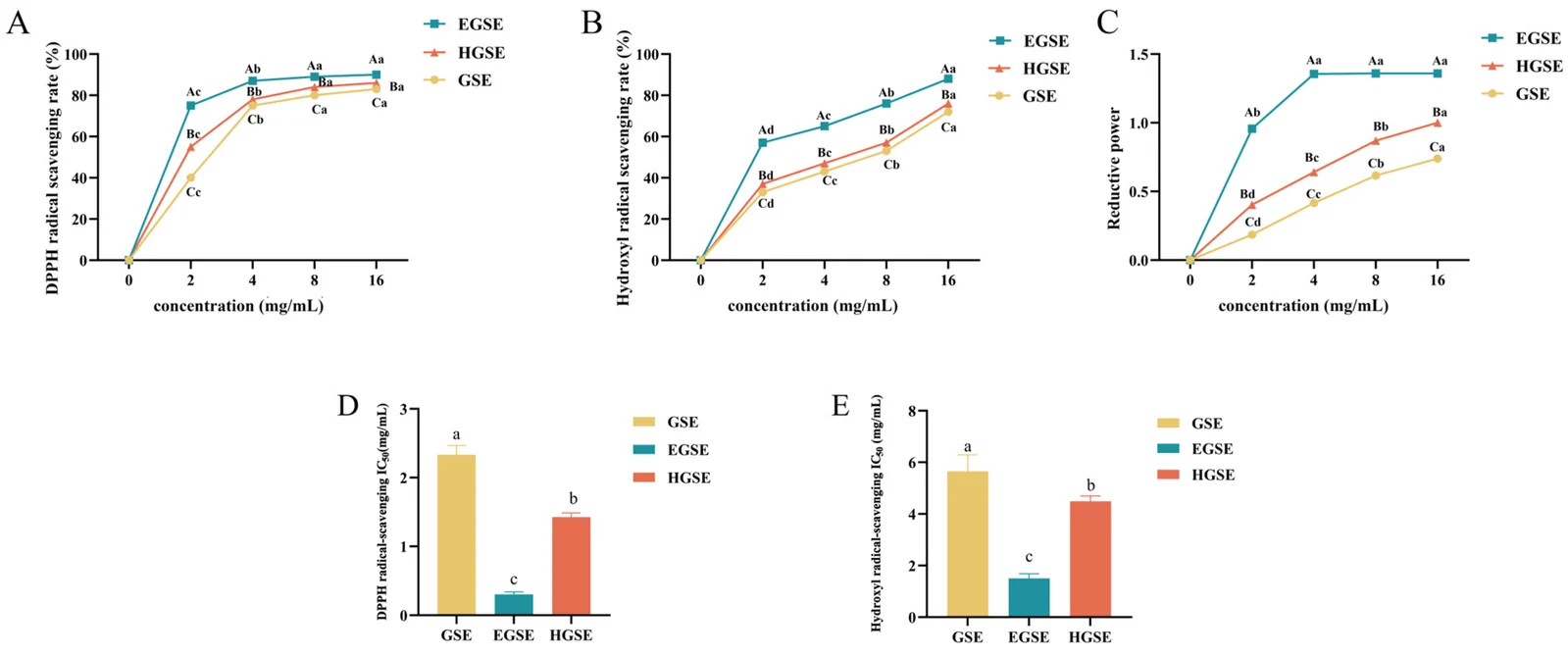
*In vitro* antioxidant activities of water extracts obtained from *G. uralensis* stems subjected to different processing treatments. (**A**) 1,1-diphenyl-2-picrylhydrazyl (DPPH) radical scavenging activity. (**B**) Hydroxyl radical scavenging activity. (**C**) Total reducing power. (**D**) DPPH radical scavenging IC_50_ values. (**E**) Hydroxyl radical scavenging IC_50_ values. Different uppercase letters indicate significant differences among different processing groups (EGSE, HGSE, and GSE) (*p* < 0.05), whereas identical uppercase letters indicate no significant difference (*p* > 0.05). Different lowercase letters indicate significant differences among different concentrations within the same treatment group (*p* < 0.05), whereas identical lowercase letters indicate no significant difference (*p* > 0.05).

All extracts exhibited concentration-dependent increases in DPPH radical scavenging activity. Across all tested concentrations, EGSE showed significantly higher scavenging activity than GSE and HGSE (*p* < 0.05), while HGSE also displayed stronger activity than GSE (*p* < 0.05). At 16 mg/mL, the scavenging rates of EGSE, HGSE, and GSE reached 90.90%, 88.56%, and 87.34%, respectively (Figure 7A). A similar pattern was observed in hydroxyl radical scavenging activity. EGSE consistently exhibited the highest scavenging capacity across all concentrations, followed by HGSE and GSE, with significant differences among the three groups (*p* < 0.05) (Figure 7B). The total reducing power assay further confirmed the superior antioxidant performance of EGSE. EGSE showed significantly higher reducing capacity than HGSE and GSE (*p* < 0.05), reaching an absorbance value of 1.75 at 4 mg/mL and approaching a plateau thereafter (Figure 7C). Although GSE and HGSE continued to increase with increasing concentrations, their reducing capacities remained lower than that of EGSE even at 16 mg/mL. In order to quantify antioxidant potency, the IC_50_ values were calculated from the dose–response curves through nonlinear regression. Consistent with the scavenging activity trends, EGSE exhibited the lowest IC_50_ values for both DPPH (0.31 ± 0.03 mg/mL) and hydroxyl radical scavenging (1.51 ± 0.17 mg/mL) (*p* < 0.05), indicating the strongest antioxidant capacity. HGSE showed intermediate IC_50_ values (1.43 ± 0.06 mg/mL for DPPH and 4.49 ± 0.20 mg/mL for hydroxyl radical) (*p* < 0.05), while GSE displayed the highestIC_50_ values (2.33 ± 0.13 mg/mL for DPPH and 5.65 ± 0.64 mg/mL for hydroxyl radical) (*p* < 0.05), reflecting the weakest activity among the three extracts (Figure 7D,E).

### 3.8. Enzymatic Processing Enhances the Protective Effects of G. uralensis Stem Extracts Against AAPH-Induced Developmental Stress

**Figure 8:**
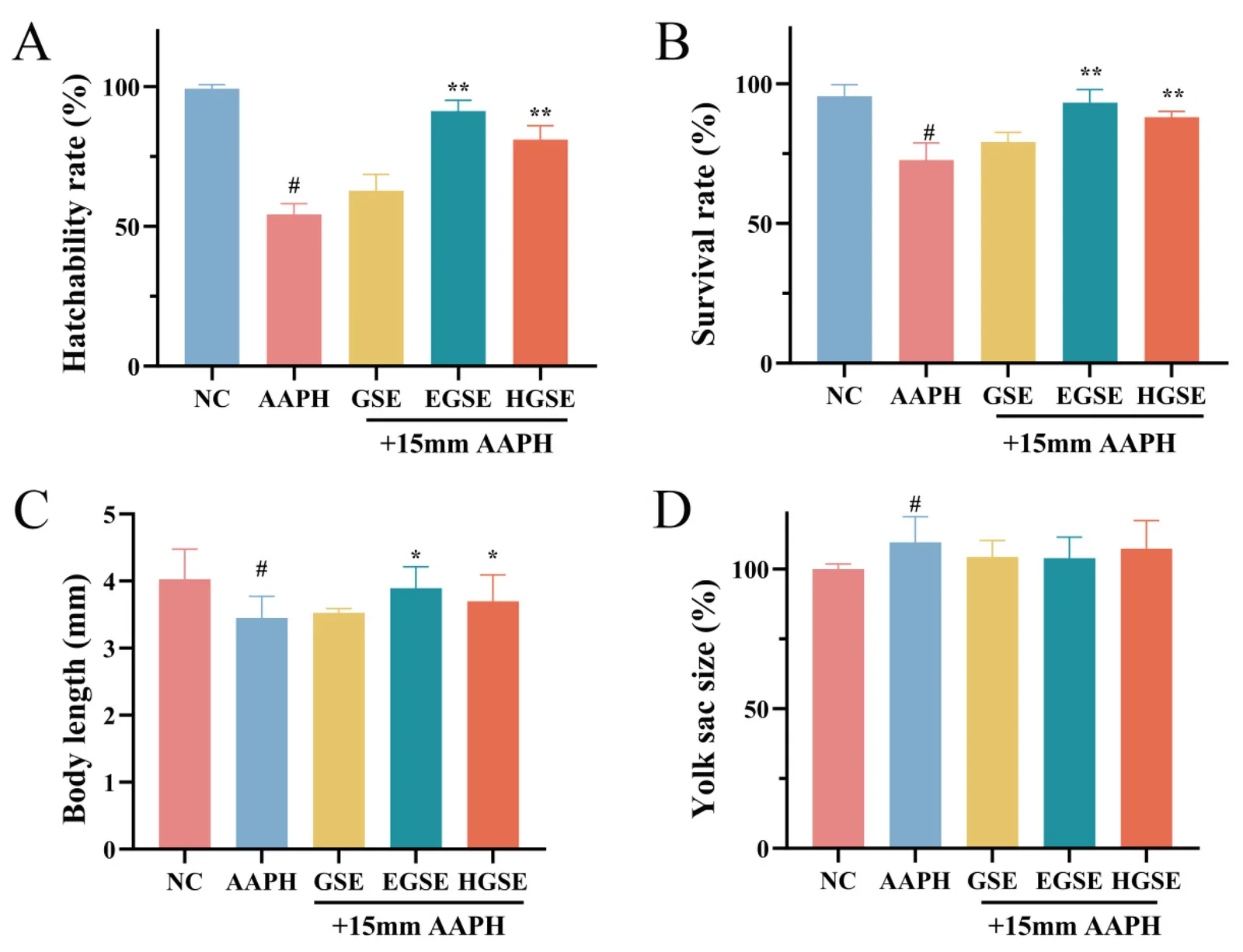
Protective effects of differently processed *G. uralensis* stem extracts against 2,2′-Azobis(2-amidinopropane) dihydrochloride (AAPH)-induced oxidative stress in zebrafish embryos. (**A**) Hatching rate. (**B**) Survival rate. (**C**) Body length. (**D**) Yolk sac area. ^#^ indicates a significant difference between negative control (NC) group and AAPH-treated group (*p* < 0.05). * and ** indicate significant differences between AAPH-treated group and extract-treated groups at *p* < 0.05 and *p* < 0.01, respectively.

To further evaluate the protective effects of the extracts in an oxidative stress-related biological model, an AAPH-induced oxidative stress model in zebrafish embryos was established to assess the effects of GSE, EGSE, and HGSE (50 μg/mL) on embryonic development and survival. The hatching rate was markedly reduced from 97.25% in the negative control (NC) group to 54.25% after AAPH exposure (*p* < 0.05). Treatment with GSE showed a limited recovery effect (59.75%, *p* > 0.05), whereas HGSE significantly restored the hatching rate to 81.00% (*p* < 0.01). Notably, EGSE exhibited the strongest protective activity, increasing the hatching rate to 91.25% (*p* < 0.01) (Figure 8A). A similar pattern was observed for embryo survival. Compared with the NC group (98.50%), AAPH treatment significantly decreased survival to 44.25% (*p* < 0.05). EGSE and HGSE supplementation significantly restored survival rates to 91.25% and 81.00%, respectively (*p* < 0.01), whereas GSE showed only a moderate and non-significant improvement (63.75%, *p* > 0.05) (Figure 8B). Morphological assessment further confirmed the protective effects of processed extracts. AAPH exposure significantly reduced embryo body length from 4.03 mm (NC) to 3.46 mm (*p* < 0.05). Both EGSE and HGSE significantly alleviated this growth inhibition, restoring body length to 3.92 mm and 3.69 mm, respectively (*p* < 0.05), while GSE showed no significant improvement (3.53 mm, *p* > 0.05) (Figure 8C). For yolk sac area, AAPH exposure resulted in a significant enlargement compared with the NC group (111.36%, *p* < 0.05), suggesting delayed nutrient utilization or developmental impairment. However, no significant differences were observed among the extract-treated groups compared with the AAPH group (*p* > 0.05), with relative yolk sac areas of 104.30%, 101.68%, and 102.15% for EGSE, HGSE, and GSE, respectively (Figure 8D). Collectively, both enzymatic and high-temperature steam processing enhanced the protective capacity of *G. uralensis* stem extracts against oxidative stress-induced developmental damage. Among them, EGSE displayed the most pronounced protective effects, consistent with its superior antioxidant activity observed *in vitro*.

### 3.9. Enzymatic Processing Preserves Antioxidant-Associated Flavonoid Signatures and Enriches Flavonoid Biosynthetic Pathways

**Figure 9:**
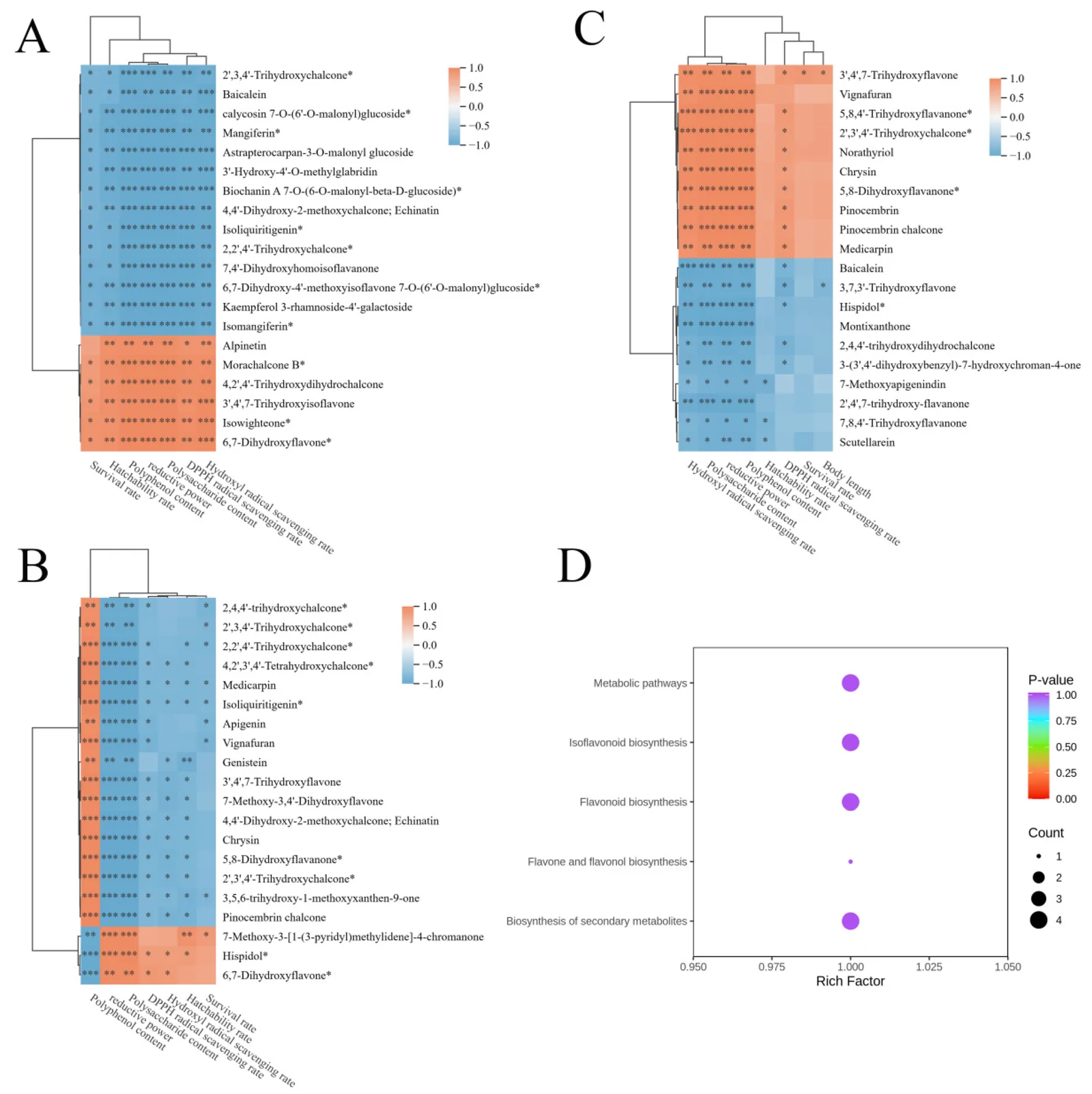
Association of key flavonoid metabolites with antioxidant activities and Kyoto encyclopedia of genes and genomes (KEGG) pathway enrichment analysis. (**A**–**C**) Correlation heatmaps showing relationships between the top 10 up- and down-regulated flavonoid metabolites (200–600 Da) and antioxidant-related parameters in (**A**) enzyme-treated *G. uralensis* stems extract (EGSE) vs. untreated *G. uralensis* stems extract (GSE), (**B**) high-temperature steam-treated *G. uralensis* stems extract (HGSE) vs. GSE, (**C**) HGSE vs. EGSE comparison groups = Red and blue represent positive and negative correlations, respectively. (**D**) KEGG pathway enrichment analysis of antioxidant-associated flavonoid metabolites. * *p* < 0.05, ** *p* < 0.01 and ns: *** *p* < 0.001.

Correlation analysis was performed between the previously identified flavonoid DEMs and antioxidant-related indices. In the EGSE vs. GSE comparison, several modified flavonoids, particularly flavanone (e.g., Alpinetin), prenylated isoflavone (such as isowighteone) and chalcone derivatives (e.g., Morachalcone B), showed strong positive correlations with *in vitro* antioxidant capacity, phenolic content, and zebrafish protective effects (cor > 0.3, *p* < 0.001. (Figure 9A). These associations identify these metabolites as candidate markers linked to the antioxidant-related characteristics of EGSE, but do not establish that they directly mediate the observed biological effects. In contrast, high-temperature steam treatment caused extensive alterations in flavonoid profiles. Several chalcone and simple flavonoid metabolites were significantly associated with polyphenol content and radical-scavenging activities; however, their correlations with hatchability and survival rates were generally weaker or less consistent than those observed for the enzyme-enriched metabolites (Figure 9B). Direct comparison of EGSE vs. HGSE further suggested that the two processing regimes were characterized by distinct flavonoid profiles that showed different patterns of association with antioxidant-related phenotypes (Figure 9C). However, these correlations should be interpreted as metabolite–phenotype associations rather than evidence that these flavonoids directly mediate the enhanced antioxidant activity of EGSE. KEGG enrichment analysis of antioxidant-associated flavonoids revealed significant enrichment in flavonoid biosynthesis, flavone and flavonol biosynthesis, and isoflavonoid biosynthesis pathways (Figure 9D). These pathways originate from common chalcone intermediates and govern the formation of the glycosylated, malonylated and hydroxylated flavonoids that were preferentially retained or enriched under enzymatic processing. However, because pathway activity was not directly measured at the transcript, protein, or enzyme-activity level, the enrichment results should be interpreted as metabolite-level pathway associations rather than evidence of pathway activation.

## 4. Discussion

Plant-derived bioactive compounds are often embedded within complex cell wall matrices, and the efficiency of their release largely depends on the extent and manner of structural remodeling during processing [5]. *G. uralensis* stems, as an underutilized plant resource, contain abundant polysaccharides, phenolics, and flavonoids, but their functional utilization is restricted by the structural recalcitrance of plant tissues. In this study, enzymatic and high-temperature steam treatments were associated with distinct structural, physicochemical, and chemical profiles in *G. uralensis* stems. Enzymatic treatment showed relatively moderate structural remodeling, whereas high-temperature steam treatment was characterized by more pronounced structural disruption and thermal-associated changes. These differences were accompanied by distinct changes in extractable phenolics, polysaccharides, flavonoid profiles, antioxidant activities, and zebrafish developmental outcomes.

Color change represents one of the most visible quality responses during plant processing and reflects a series of complex chemical transformations. Thermal conditions can accelerate non-enzymatic browning reactions, including phenolic oxidation, Maillard reactions between carbonyl and amino compounds, and polyphenol polymerization, leading to the formation of brown pigments [26]. Previous studies have demonstrated that high-temperature extraction or roasting of *G. uralensis* induces pronounced browning, which is closely associated with temperature-dependent Maillard reactions and phenolic transformation [27,28]. In the present study, enzymatic treatment caused minimal alterations in the appearance of *G. uralensis* stems, whereas high-temperature steam treatment resulted in substantial darkening, suggesting enhanced thermal-induced browning reactions. Such heat-associated color transformation may not only affect sensory quality but also indicate potential oxidation or structural modification of phenolic compounds, thereby influencing the functional properties of processed extracts.

Cell wall remodeling represents a critical determinant governing the accessibility and release of plant-derived bioactive compounds [29]. In *G. uralensis* stems, cellulose, hemicellulose, and pectin form a complex matrix that physically restricts the diffusion of phenolics, flavonoid glycosides, and polysaccharides [30]. Enzyme-assisted processing provides a targeted strategy to overcome this structural barrier, as pectinase-mediated degradation of pectic polysaccharides can weaken cell wall cohesion and enhance the exposure of intracellular components [11,12]. In this study, enzymatic treatment induced moderate structural loosening rather than extensive tissue destruction, accompanied by increased microstructural complexity and improved extraction efficiency of phenolics and soluble polysaccharides. These findings suggest that enzymatic processing may promote bioactive compound accessibility through controlled cell wall modification rather than nonspecific structural disruption, consistent with previous reports in plant-derived materials [31,32]. In contrast, high-temperature steam treatment relies on thermal and hydrothermal effects to disrupt plant matrices. The combined action of heat, moisture, and pressure can accelerate cell wall rupture and increase structural porosity [33,34]; however, these effects are often accompanied by non-selective physicochemical transformations, including polysaccharide depolymerization, crystalline rearrangement, and degradation of heat-sensitive compounds. Although thermal processing promoted structural disruption and altered polysaccharide composition, the reduction in phenolic retention suggests that excessive thermal stress may compromise the preservation of functional constituents [6]. Therefore, greater structural destruction does not necessarily translate into enhanced bioactive compound availability.

Polysaccharide remodeling further highlighted the distinct regulatory mechanisms of the two processing strategies. Enzymatic treatment mainly improved polysaccharide extractability with limited changes in monosaccharide composition, indicating a relatively mild and controlled modification of the polysaccharide network [35,36]. Conversely, high-temperature steam treatment caused pronounced monosaccharide redistribution, characterized by increased acidic sugar fractions, suggesting more extensive hydrothermal degradation and transformation of structural polysaccharides [37,38]. Collectively, these results suggest that enzymatic processing achieves a favorable balance between matrix disruption and functional component preservation, whereas high-temperature steam treatment induces broader molecular rearrangement that may alter the integrity and functionality of native polysaccharides [39].

Flavonoids are considered major contributors to the antioxidant properties of *G. uralensis* due to their diverse hydroxylation patterns, glycosylation modifications, and strong radical-scavenging capacity [40,41,42]. However, the abundance, structural characteristics, and bioavailability of flavonoids are highly susceptible to processing conditions. In the present study, enzymatic and high-temperature steam treatments produced distinct flavonoid profiles, indicating that processing conditions were associated with different patterns of flavonoid remodeling. The flavonoid alterations induced by enzymatic treatment may primarily result from enhanced accessibility and redistribution of bound flavonoids following cell wall degradation [43]. Many plant flavonoids exist in conjugated forms, associated with cell wall polysaccharides or other macromolecules through glycosidic or ester linkages, which limits their extractability [30,44]. Enzyme-mediated matrix loosening may facilitate the release and redistribution of bound flavonoid forms, potentially contributing to the altered flavonoid composition observed after enzymatic treatment. However, the present untargeted metabolomic data do not allow us to determine whether these changes correspond to increases in the absolute concentrations or biological activities of individual flavonoids. In contrast, high-temperature steam treatment induced a more complex flavonoid transformation pattern [45,46]. Although thermal disruption of plant matrices may enhance the release of certain compounds, elevated temperatures can simultaneously promote flavonoid oxidation, degradation, isomerization, or polymerization [45], particularly for polyhydroxylated flavonoids that are sensitive to thermal stress [47]. Consequently, thermal processing may generate new flavonoid derivatives or increase certain simple flavonoids, but these chemical changes do not necessarily translate into enhanced antioxidant functionality [48]. This suggests that flavonoid structural features and stability may be more critical determinants of bioactivity than overall abundance alone. Furthermore, correlation analysis identified several flavonoids that were significantly associated with antioxidant-related measurements and zebrafish developmental outcomes. These metabolites may therefore represent candidate compounds for further investigation. KEGG enrichment further indicated that the associated metabolites were distributed among flavonoid biosynthesis, flavone and flavonol biosynthesis, and isoflavonoid biosynthesis categories. These results provide a metabolite-level framework for understanding the chemical differences among processing groups.

Several limitations should be acknowledged in this study. First, although enzymatic and high-temperature steam treatments showed distinct effects on the functional properties of *G. uralensis* stems, further optimization and evaluation of their industrial feasibility are needed. In addition, an enzyme-free control subjected to the same incubation and drying conditions was not included; therefore, the effects of pectinase cannot be completely distinguished from those of the thermal incubation and drying processes. Second, the pectin content and detailed compositional characteristics of the raw *G. uralensis* stems were not directly quantified, which limited a more precise evaluation of the substrate basis for pectinase treatment. Furthermore, furfural was not determined, limiting the assessment of potential thermal degradation products during high-temperature steam treatment. Third, untargeted metabolomic and correlation analyses identified several flavonoid metabolites associated with antioxidant-related parameters; however, targeted quantification of individual flavonoids and compound-specific functional validation were not performed. Therefore, differences in flavonoid relative abundance should not be interpreted as evidence of absolute enrichment or direct causal effects on antioxidant activity. Future studies integrating targeted quantification, structural characterization, and compound-specific functional assays will be necessary to clarify the contribution of key flavonoids to the biological effects of processed extracts. Moreover, although IC_50_ values were determined for DPPH and hydroxyl radical-scavenging activities, no antioxidant reference standards were included for direct comparison. Antioxidant activities were evaluated based on extract concentrations without normalization to standardized antioxidant equivalents, which may affect comparisons among processing groups. Lastly, although the AAPH-induced zebrafish model demonstrated protective effects of the processed extracts against developmental stress, oxidative stress-related biomarkers, such as reactive oxygen species (ROS), lipid peroxidation, or endogenous antioxidant enzyme activities, were not directly measured. Thus, the observed improvements in survival, hatching, and body length should not be interpreted as definitive evidence of antioxidant mechanisms. Other biological effects, including general developmental or nutritional effects, may also contribute to the observed phenotypes.

## 5. Conclusions

This study demonstrates that enzymatic and high-temperature steam treatments induce distinct structural and metabolic remodeling patterns in *G. uralensis* stems. Enzymatic treatment increased extract solubility from 0.25% to 0.32%, soluble polysaccharide content from 266.06 ± 1.58 to 319.40 ± 4.19 mg/g, and total phenolic content from 49.69 ± 0.65 to 63.99 ± 0.81 mg/g, whereas high-temperature steam treatment decreased solubility to 0.14% and total phenolic content to 38.39 ± 0.52 mg/g. In parallel with these compositional differences, EGSE exhibited the highest DPPH radical-scavenging activity (90.90% at 16 mg/mL) and showed stronger protection of zebrafish embryo survival and hatching under AAPH-induced oxidative stress, with both rates reaching 91.25%. Overall, enzymatic treatment showcased greater potential for improving the extractable physicochemical and antioxidant-related properties of *G. uralensis* stems under the conditions investigated, while the observed associations between processing, metabolite profiles, and biological effects warrant further targeted and mechanistic validation.

## Figures and Tables

**Table 1:** Monosaccharide composition and molar percentage (mol%) from *G. uralensis* stems under different treatment conditions.

Monosaccharide Composition and Molar Percentage (%)	Untreated *G. uralensis* Stems (GSE)	Enzyme-Treated *G. uralensis* Stem Extract (EGSE)	High-Temperature Steam-Treated *G. uralensis* Stemextract (HGSE)
Glucose	76.64	71.07	46.64
Galactose	5.82	6.4	6.85
Arabinose	4.68	4.77	15.53
Galacturonic acid	3.49	5.87	20.58
Xylose	3.27	3.52	1.46
Mannose	2.08	2.99	2.46
Rhamnose	1.3	2.07	3.33
Glucuronic acid	1.04	1.14	1.05
Glucosamine	0.69	0.87	—
Ribose	0.38	0.4	1.06
Fucose	0.27	0.56	1.03
Guluronic acid	0.16	0.14	—
N-acetylgalactosamine	0.15	0.21	—
Mannuronic acid	—	0.02	—

## Data Availability

The original contributions presented in this study are included in the article/Supplementary Materials. Further inquiries can be directed to the corresponding authors.

## Supplementary Materials

The following supporting information can be downloaded at: https://www.mdpi.com/article/10.3390/foods15183315/s1, Table S1: Information on reagents and instruments.
