# Supplementary material for: Comparative Insights into Enzymatic and High-Temperature Steam Treatment of Glycyrrhiza uralensis Fisch. Stems: Structural Remodeling, Flavonoid Transformation and Antioxidant Enhancement
Source: Foods. 2026 Sep 19;15(18):3315. doi: 10.3390/foods15183315 (PMC13606405; doi:10.3390/foods15183315)
Supplement: Supplementary file 1 [file foods-15-03315-s001.zip › foods-4525968-supplementary.pdf]

Table S1 Information on reagents and instruments.

Item	Model/Series	Manufacturer	Country
**Reagents**			
Pectinase	—	Beijing Solarbio Science & Technology Co., Ltd.	China
1-Phenyl-3-methyl-5-pyrazolone (PMP) methanol	—	Merck KGaA	Germany
Acetonitrile	—	Merck KGaA	Germany
Formic acid	—	Merck KGaA	Germany
2,2-Diphenyl-1-picrylhydrazyl (DPPH)	—	Sinopharm Chemical Reagent Co., Ltd.	China
Folin-Ciocalteu reagent	—	Sinopharm Chemical Reagent Co., Ltd.	China
Gallic acid	—	Sinopharm Chemical Reagent Co., Ltd.	China
Glucose	—	Sinopharm Chemical Reagent Co., Ltd.	China
Sodium carbonate	—	Sinopharm Chemical Reagent Co., Ltd.	China
2,2′-Azobis(2-amidinopropane) dihydrochloride (AAPH)	—	Sigma-Aldrich	USA
Potassium Bromide (KBr)	—	Sinopharm Chemical Reagent Co., Ltd.	China
Trichloroacetic acid	—	Sinopharm Chemical Reagent Co., Ltd.	China
Potassium ferricyanide	—	Sinopharm Chemical Reagent Co., Ltd.	China
Phosphate Buffered Saline (PBS)	—	Sinopharm Chemical Reagent Co., Ltd.	China
**Instruments**			
Multilayer polyethylene fermentation bags	—	Wenzhou Tangxing Packaging Co., Ltd.	China
D65 standard light cabinet	P60	TILO	China
Industrial camera	DS-2CD2T	Hangzhou Hikvision Digital Technology Co., Ltd.	China
Temperature-controlled incubator	IN160	Memmert	Germany
Electric Thermostatic Blow Dryer	DHG-9070A	Shanghai Yiheng Scientific Instrument Co., Ltd.	China
Centrifuge	TG16-WS	Hunan Xiangyi Laboratory Instrument Development Co., Ltd.	China
Rotary evaporator	RE-52AA	Shanghai Yarong Biochemical Instrument Factory	China

High-pressure steam sterilizer	LMQ.C-80E	Shandong Xinhua Medical Instrument Co., Ltd.	China
Scanning electron microscope (SEM)	JSM-IT510	JEOL Ltd.	Japan
Thermogravimetric analyzer (TGA)	Q50	TA Instruments	USA
X-ray diffractometer (XRD)	MiniFlex 600	Rigaku Corporation	Japan
Fourier-transform infrared spectrometer (FTIR)	Nicolet iS50	Thermo Fisher Scientific	USA
Microplate reader	BioTek Epoch	BioTek Instruments, Inc.	USA
Stereomicroscope	SZ61	Olympus	Japan
Ultra-performance liquid chromatography system	ExionLC™ AD	SCIEX	USA
Triple quadrupole linear ion trap mass spectrometer	QTRAP 6500+	SCIEX	USA
Agilent SB-C18 column	880975-902	Agilent Technologies	USA
